# ﻿*Austropallenehalanychi* sp. nov., a new species of sea spider (Pycnogonida, Callipallenidae) from the Ross Sea, Antarctica

**DOI:** 10.3897/zookeys.1185.108286

**Published:** 2023-11-28

**Authors:** Jessica R. Zehnpfennig, Andrew R. Mahon

**Affiliations:** 1 Department of Biology, Central Michigan University, Mount Pleasant, MI, USA Central Michigan University Mount Pleasant United States of America

**Keywords:** Biodiversity, morphology, mtDNA, phylogeny, taxonomy

## Abstract

Here we present *Austropallenehalanychi***sp. nov.**, a new species of pycnogonid within the family Callipallenidae (Pycnogonida), collected from the Ross Sea, Antarctica. While retaining key morphological features known for the genus *Austropallene* Hodgson, 1915a, the new species is distinguished from congeners by its much larger size, along with the combined absence of a denticle on the inner surface of the fixed finger of the chelifore claw along with the presence of small conical outgrowths where the fixed finger of the chelifore claw meets the movable finger on both the dorsal and ventral sides, and also the ability to fully close the chelifore claw. Additionally, the complete mitochondrial genome of *A.halanychi* is consistent with other members of the genus *Austropallene* in terms of gene order and directionality. A phylogenetic tree consisting of mitochondrial protein-coding gene data places *A.halanychi* as sister to *Austropallenecornigera* (Möbius, 1902). Additionally, a phylogenetic tree constructed using partial COI data from other callipallenids placed the new species in a clade containing the genus *Austropallene*. The combination of molecular data in addition to key morphological differences from similar species in the genus leaves no doubt that the new taxon is a new Antarctic species of *Austropallene*.

## ﻿Introduction

The family Callipallenidae (Pycnogonida, Arthropoda) is represented globally by 17 genera and over 200 species and subspecies (Bamber et al. 2023). Representatives of the family are highly variable in size, color, and form but are characterized by the presence of functional chelifores and ovigers in both males and females, and with compound spines being present on the ovigers (Arango and Brenneis 2013). The genus *Austropallene* Hodgson, 1915a was created for a species that did not fit into any of the previously established genera within Callipallenidae, including *Pseudopallene* Wilson, 1878 and *Cordylochele* Sars, 1888. The original description of the genus *Austropallene*Hodgson (1915a) is listed by Fry and Stock (1978) in their bibliography as “Hodgson (1914)”. Following the creation of *Austropallene*, Hodgson (1915b) more fully described the genus and several species. Hodgson’s original description of *Austropallene* is as follows:

“*A genus established to include those forms which Mobius, Prof. Bouvier, and the present writer have included in different genera. The presence of cephalic spurs is a most noticeable feature and is confined to all these southern species. Body robust or slender, segmentation distinct, lateral processes close together or widely separated. Large and stout cephalic spurs. Eyes well developed. Proboscis tapering, with or without a setose wreath. Cheliferi stout, chelae short and powerful. Palps no trace. Ovigers 10-jointed, without a terminal claw. In the male a distal swelling on the fifth joint. No auxiliary claws.*” (Hodgson 1915a: 161)

Subsequently three species, originally placed within *Pseudopallene*, were included in *Austropallene*: *A.cornigera* (Möbius, 1902), *A.cristata* (Bouvier, 1911), and *A.spicata* Hodgson, 1915a. Despite Hodgson citing *Cordylochele* Sars, 1888, no *Cordylochele* species were moved to *Austropallene* (Hodgson 1915a,1915b). Subsequently, *A.spicata* was determined by Calman (1915) to be synonymous with Bouvier’s previously described species, *A.brachyura* Bouvier, 1911 and, in addition, he described *A.tibicina* Calman, 1915 and clarified that “[…] in *Austropallene* there is usually, perhaps always, a minute terminal spine, if not a ‘claw’, in the ovigers” (Calman 1915: 38). *A.calmani* Gordon, 1944 and *A.gracilipes* Gordon, 1944 and the smallest member of the genus, *A.tcherniai* Fage, 1952, were described later and then the genus was redescribed by Pushkin in 1993, with the addition of three new species: *A.tenuicornis* Pushkin, 1993, *A.spinicornis* Pushkin, 1993, and *A.bucera* Pushkin, 1993. Apart from *A.lukini* Turpaeva, 2002, found in the northern Sea of Okhotsk, the ten other species within *Austropallene* described to date are restricted to Antarctic waters.

Within Pycnogonida, 26 complete or nearly complete mitochondrial genomes have been reported (Hassanin et al. 2005; Podsiadlowski and Braband 2006; Park et al. 2007; Masta et al. 2010; Dietz et al. 2011; Carapelli et al. 2013; Zehnpfennig et al. 2022), including two belonging to the genus *Austropallene*: *A.cornigera* and *A.bucera* (Suppl. material 1). Mitochondrial genomes contain a moderate amount of gene order conservation among related taxa and arrangements in gene order are often used for phylogenetic inferences (Boore and Staton 2002; Varney et al. 2021; Zehnpfennig et al. 2022). Published pycnogonid mitochondrial genomes have a conserved protein-coding gene order that contains the standard compliment of bilaterian mtDNA genes (i.e. 13 protein-coding genes, 2 ribosomal RNA genes, and 22 tRNAs (Boore 1999; Vallès et al. 2008). Furthermore, Zehnpfennig et al. (2022) found that the order and directionality of protein-coding genes found in pycnogonids are identical in each family as well as in other chelicerates (e.g. *Limuluspolyphemus*; Lavrov et al. 2000). The order of tRNA genes, however, differ by families, with representatives of Nymphonidae and Callipallenidae having distinct tRNA arrangements, exclusive to members within those families.

In this study, we describe *Austropallenehalanychi* sp. nov., a new species from the Ross Sea, Antarctica (Southern Ocean). The description of this novel species is supported by morphological characteristics as well as phylogenetic analyses conducted using data from its complete mitochondrial genome. The combination of morphology and molecular data support the distinction of this new species from other described species within the genus.

## ﻿Materials and methods

### ﻿Study site and sample collection

**Figure 1.**
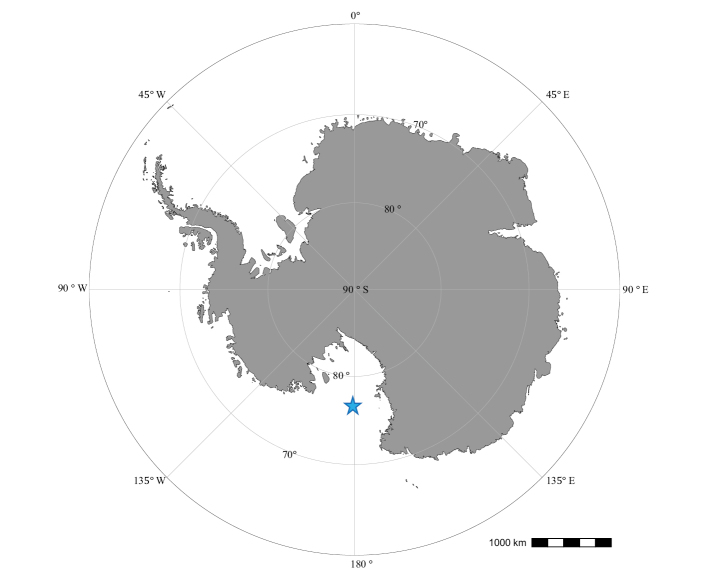
*Austropallenehalanychi* sp. nov., collection location (blue star) within the Ross Sea, Antarctica.

A single specimen of *Austropallenehalanychi* sp. nov. was collected via Blake trawl while aboard the RVIB *Nathaniel B. Palmer* (NBP12-10) on 31 January 2013 as part of a multi-institutional research expedition involving Auburn University and Central Michigan University to investigate the genetic connectivity and biogeographic patterns of Antarctic benthic invertebrates. The specimen was collected in Antarctica from the Ross Shelf (Ross Sea) at 560 m depth (75°19'46.7"S, 176°59'06.3"W, Fig. 1). Upon collection, the specimen was identified to genus and preserved in ~95% ethanol until it could be returned to the laboratory for further investigation. A map of the study site (Fig. 1) was created using the maps package in R (R Core Team 2022). The specimen was donated to the Smithsonian National Museum of Natural History (NMNH) invertebrate zoology collection under accession number 1548440.

### ﻿Morphological identification

The specimen was identified to the genus level by consulting the original description of *Austropallene* by Hodgson (1915a, 1915b) as well as the description by Pushkin (1993), in the most recent review of *Austropallene*. Species-level identification was attempted using the available morphological keys as well as the original descriptions of closely related species (Hodgson 1915a, 1915b; Pushkin 1993; Child 1995). This revealed that the specimen does not belong to any described species of *Austropallene*. Stereomicroscopic images of the specimen were taken with a Leica M165C, with LAS v. 4.3 software. Measurements of structures were determined using the optical micrometer provided with the LAS software.

### ﻿Molecular methods

Genomic DNA was extracted from the specimen’s leg muscle tissue with the Qiagen DNEasy Blood and Tissue Kit (Qiagen, Inc., Valencia, CA) according to the manufacturer’s recommendations. Extracted DNA was submitted to the RTSF Genomics Core at Michigan State University for library preparation and sequencing. The sample (in addition to other samples) was sequenced with a SP 300 cycle flow cell and Illumina NovaSeq with PE150 v2 chemistry.

### ﻿Data analyses

Paired-end sequences were quality checked using FASTQC (Andrews et al. 2010) and assembled with NOVOPlasty v. 4.3.1 (Dierckxsens et al. 2016) using the previously published whole mitochondrial genome of *Acheliabituberculata* Hedgepeth, 1949 (Ammotheidae) as a reference sequence (Park et al. 2007). The assembly was screened with BLAST (Altschul et al. 1990) to determine if the resulting contig hit (with the highest percent query cover) to a previously published pycnogonid mitochondrial genome. This was used as a baseline for mitochondrial DNA identification. The contig containing the mitochondrial genome was annotated with the MITOS web server (Bernt et al. 2013) and gene boundaries were checked manually in Artemis v. 17.0 (Rutherford et al. 2000). Total G-C content (as percent total DNA) was determined using Quast v. 5.0 (Gurevich et al. 2013) on the assembled mitochondrial genome. Gene length, directionality, and order for the assembled mitochondrial genome were also assessed and summarized (Suppl. material 2).

Protein-coding gene sequences from the resulting mitochondrial genome, as well as complete or nearly complete mitochondrial genomes belonging to pycnogonids available from publicly available repositories (e.g. GenBank), partial mitochondrial gene data for individuals belonging to the family Ascorhynchidae, and mitochondrial protein-coding gene data published for *Limuluspolyphemus* (as an outgroup) were compiled for phylogenetic reconstruction. To create a phylogeny containing representatives from all known sea spider families, partial mitochondrial gene data were used to represent Ascorhynchidae as there are no currently available complete mitochondrial genomes belonging to this family. Nucleotide sequences from protein-coding genes were aligned using MAFFT (Katoh et al. 2002). TrimAL was used to trim protein-coding genes for each family (Capella-Gutiérrez et al. 2009). A concatenated matrix of aligned and trimmed protein-coding genes was created using FASconCAT v.1.0 (Kück and Meusemann 2010). Phylogenetic analyses were conducted using IQtree2 (Minh et al. 2020) with a partitioned maximum likelihood analysis using the best-fitting model for each gene resulting in a phylogeny of sea spider families. The resulting topology was evaluated based on 1000 rapid bootstrap replicates. To further investigate the placement of *A.halanychi* sp. nov., an additional phylogenetic analysis utilizing partial COI sequences available for members of the family Callipallendiae was conducted with COI data from *Nymphonaustrale* Hodgson, 1902 as an outgroup using the aforementioned methods. (Suppl. material 1). To visualize our resulting phylogenetic trees, we used FigTree v. 1.4.4 (Rambaut and Drummond 2012). MEGA v. 11 (Tamura et al. 2021) was used to assess genetic distance (using uncorrected *p*-distance) between the protein-coding genes and ribosomal RNA genes from *A.halanychi* sp. nov. and the already published genomes of *A.cornigera* and *A.bucera* (Zehnpfennig et al. 2022). The protocol for mitochondrial genome assembly and annotation is available at: https://www.protocols.io/view/mitochondrial-genome-assembly-and-annotation-cvfgw3jw.

## ﻿Results

### ﻿Systematics

**Class Pycnogonida Latreille, 1810**

**Order Pantopoda Gerstäcker, 1863**

**Family Callipallenidae Hilton, 1942**

**Genus *Austropallene* Hodgson,1915**

#### ﻿ Austropallene halanychi sp. nov.

Taxon classificationAnimaliaPantopodaCallipallenidae

5EAAF589-7675-53BE-AC7C-C7C05918669D

https://zoobank.org/C15B8CD5-0E11-47C4-8626-81D33D718055

Figs 2 , 3 , 4

##### Type locality.

Antarctica, Ross Sea, Ross Shelf, 570 m depth, 75°19'46.7"S, 176°59'06.3"W, collected via benthic trawl, 31 January 2013, RVIB *Nathaniel B. Palmer* (NBP12-10), A. Mahon leg.

##### Type specimen.

Holotype male preserved in ethanol, original label “Antarctica, Ross Sea, Ross Shelf, 570 m depth,75°19'46.7"S, 176°59'06.3"W, 31 January 2013, J. Zehnpfennig and A. Mahon”, NMNH 1548440”; handwritten label “Ch 226.1E”.

##### Diagnosis.

The new species can be differentiated from all other described *Austropallene* species based on its much larger size, as well as its unique and distinctive chelifores. The chelifore claw of *A.halanychi* sp. nov. closes completely when the fingers converge, with no visible space remaining between the fixed and movable fingers. The movable finger of the chelifore claw is distinctly shorter than the fixed finger, and the chelifore fingers do not contain any denticles or notches on their inner surfaces.

##### Description of holotype (male).

**Figure 2.**
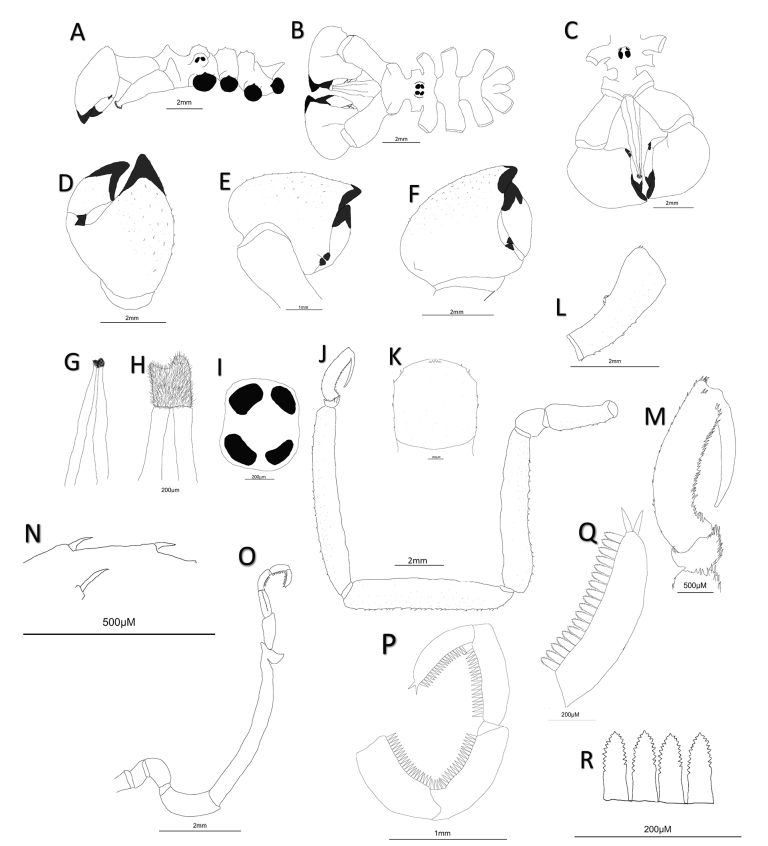
*Austropallenehalanychi* sp. nov., male holotype **A** side view (scale) **B** dorsal view **C** front view, note cephalic spurs **D** dorsal view of opened chelifore claw; note absence of denticle and conical outgrowth at base of fixed and movable fingers **E** dorsal view of chelifore claw **F** ventral view of chelifore claw; note conical outgrowth **G** dorsal view of proboscis with setose wreath H setose wreath around proboscis **I** dorsal view of eyespot **J** third walking leg **K** first coxa of third walking leg **L** side view of second coxa of third walking leg **M** propodus of walking leg **N** tubercles and spines on walking legs **O** ovigerous leg of male **P** strigilis of ovigerous leg **Q** 10^th^ segment of oviger; note terminal spines **R** serrated ovigerous leg spines (compound spines) on strigilis of ovigerous leg.

**Figure 3.**
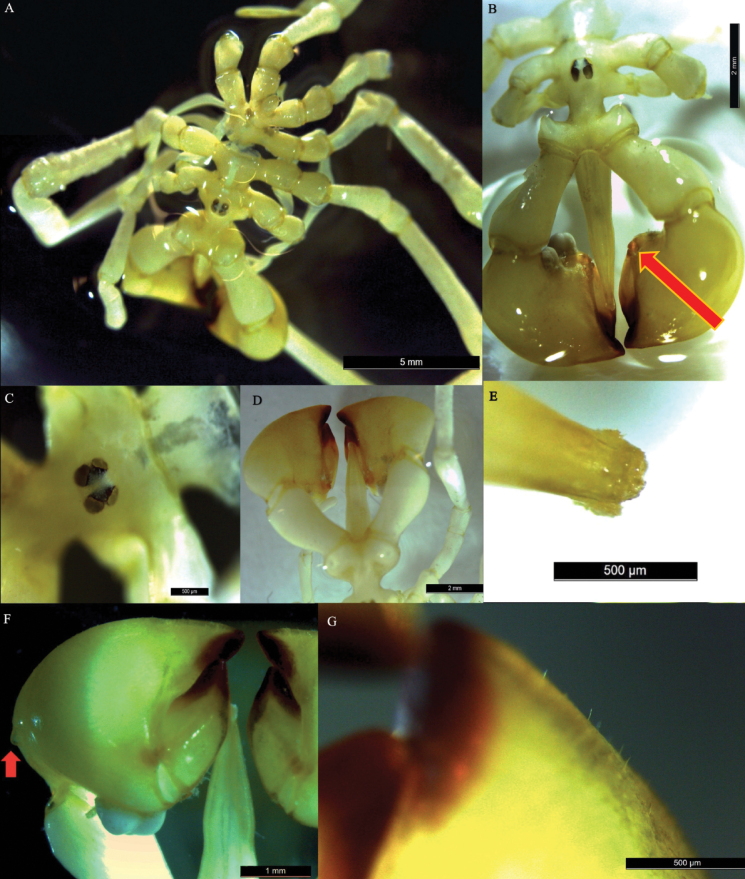
*Austropallenehalanychi* sp. nov., male holotype **A** dorsal view **B** dorsal-frontal view; note shape, relative size, and black tips of chela fingers, cephalic spurs, and eye tubercle and eyes; note sharp conical outgrowths at base of fixed and movable fingers of chelifores (red arrow) **C** eye tubercle, top view **D** dorsal view of cephalic spurs, chelifore claws, and proboscis; note setose wreath on proboscis **E** setose wreath of proboscis **F** dorsal view of chelifore claws; note lack of space between chelifore fingers and small outgrowth (red arrow) **G** dorsal view of tip of chelifore claw; note presence of bristles along top of immovable finger.

Slender appearance, fully segmented trunk; neck distinct, large oblong ovigers attaching laterally; cephalic spurs present (Figs 2A–C, 3A, B). Ocular tubercle (Figs 2A–C, 3B, C) low, rounded, slightly inclined backwards, prominent distal papillae; four darkly pigmented eyes, anterior pair larger than posterior pair (Fig. 2A, I). Lateral processes long, smooth, without spines. Abdomen short, conical, swollen distally, cleft anal opening. Proboscis (Figs 2A–C, 3B, D, E) directed ventrally, broad at base, conical, slight mid-point constriction, distally tapering; mouth surrounded by setose wreath (Figs 2G, H, 3E).

Chelifore (Figs 2D–F, 3B, D, F, G), scape large and oblong, shorter than proboscis, directed slightly anteroventrally, slight tapering towards base; chelae long, 77.5% of scape length; tips of chela fingers blackened; fingers longer than one-half of palm length, fixed finger of chelifore concave with pointed tip. Movable finger 0.73 mm shorter than fixed finger, convex, with rounded tip. Chelifore fingers converge completely, no space present between fingers, denticles not present on inner surface of either finger. Two small conical outgrowths present where fingers of chelifore claw meet (Fig. 3B, arrow). Chelifore scape and claw contain small setae along dorsal and ventral sides.

**Figure 4.**
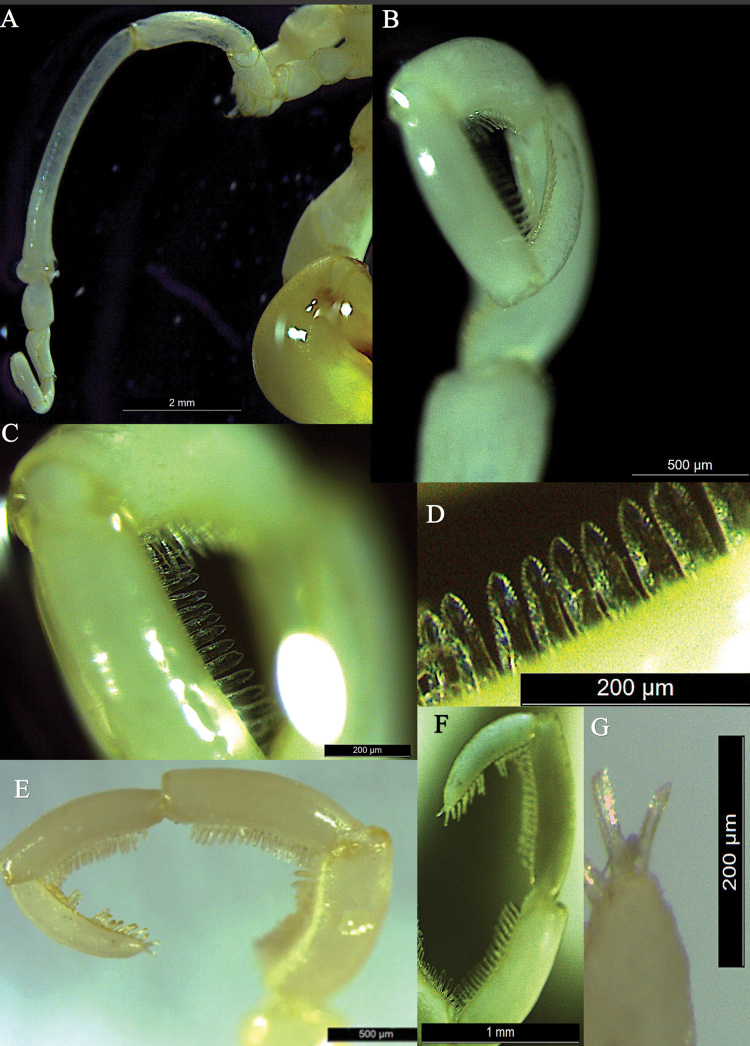
*Austropallenehalanychi* sp. nov., male holotype **A** oviger, entire **B** strigilis, close-up view showing compound spines on segments 7–10 **C** compound spines on segment 9 **D** compound spines on segment 9 **E** strigilis, side view, showing compound spines on segments 7–10 (some spines on segment 10 are damaged or missing) **F** segments 8–10 with compound spines, and segment 10 with two terminal spines **G** segment 10, top view, close-up, with two terminal compound spines.

Oviger 10-segmented (Figs 2O–R, 4A–G), fifth segment longest, slightly curved, with prominent apophysis distally; oviger compound spines serrated, present on terminal four segments of the strigillis, strigilis formula: 20:20:18:18, with 2 prominent terminal spines. Some of the spines are broken off (Fig. 4E, F).

Walking legs (Fig. 2J–N), long, slender, sparse short spinules dorsally and ventrally on major segments; small conical tubercles and setae on first coxa. Femur shortest leg segment; tibia 2 longest segment; tarsus short, curved, small distal spine in line with heel spines; propodus curved, distinct propodal heel; five heel spines, relatively similar in size; many sole spines present; auxiliary claws absent, main claw extends nearly to heel. Gonopores and cement glands not observed.

##### Measurements (male holotype-mm).

Body length (anterior end of cephalon to posterior end of trunk) = 10.50; body width (right lateral process of leg 1 + width of trunk + left lateral process of leg 1) = 4.02; trunk width = 1.24; abdomen length = 6.97; ocular tubercle height = 0.39; proboscis length = 2.73; chela fingers = 2.61; main claw length = 3.37; scape = 2.39; oviger 5^th^ segment = 4.18; 10^th^ segment = 0.74; terminal oviger spines = 0.10; leg span 63.84mm (distance between terminal claws second pair of walking legs); walking legs 29.78 mm in length (from where first coxa meets lateral process to terminal claw on second pair of walking legs); 3^rd^ walking leg coxa 1 = 1.23, coxa 2 = 2.60, coxa 3 = 1.17, femur = 6.69, tibia 1 = 6.85, tibia 2 = 7.89, tarsus = 0.32, propodus = 1.95, terminal claw = 1.39.

##### Etymology.

The species (*halanychi*, male genitive) is dedicated to Dr Kenneth M. Halanych, a mentor, colleague, and prolific marine invertebrate scientist whose commitment and dedication to the benthic marine systems in the Southern Ocean has provided a wealth of information related to biodiversity in the Antarctic system.

## ﻿Discussion

### ﻿Molecular evidence

**Figure 5.**
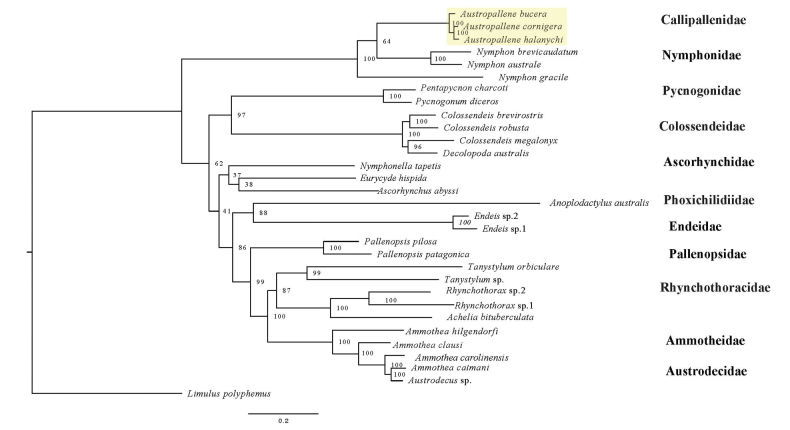
Phylogenetic tree constructed with protein-coding gene data. The phylogenetic tree shows placement of *Austropallenehalanychi* sp. nov. and was constructed using protein-coding gene data with IQ-TREE2; it uses a maximum likelihood approach and best-fit model (GTR+F+R5). The clade containing *A.halanychi* sp. nov. is highlighted, and bootstrap support (based on 1000 rapid bootstrap replicates) is included.

The complete mitochondrial genome of *Austropallenehalanychi* sp. nov. is 15,022 bp in length, and has a G-C content of 21.10% (Table 1) and contains 13 protein-coding genes, 22 tRNA genes, and 2 rRNA genes, along with an AT-rich control region, consistent with the other complete published pycnogonid mitochondrial genomes (Hassanin et al. 2005; Podsiadlowski and Braband 2006; Park et al. 2007; Masta et al. 2010; Carapelli et al. 2013; Zehnpfennig et al. 2022). The order and directionality (strand placement) of all the genes in the mitogenome of *A.halanychi* were found to be consistent with the two previously published mitogenomes belonging to *A.bucera* and *A.cornigera* (Suppl. material 2) (Zehnpfennig et al. 2022). The observed gene pattern follows the same gene pattern seen in representatives of Callipallenidae (*A.cornigera* and *A.bucera*) and Nymphonidae, which shows rearrangements in trnA (Alanine), trnE (Glutamic acid), trnY (Tyrosine), trnP (Proline), trnS1 (Serine), trnV (Valine), trnQ (Glutamine), trnM (Methionine), and trnI (Isoleucine) when compared to species belonging to other families in Pycnogonida (Suppl. material 2). The phylogenetic tree constructed with protein-coding gene data recovered a monophyletic clade containing *A.halanychi* as sister to *A.cornigera* (BS = 100), with this grouping placed as sister to *A.bucera* (BS = 100) (Fig. 5). Genetic distances calculated using uncorrected *p*-distances supported the findings in the phylogenetic tree, with all the protein-coding genes (excluding ND3) and ribosomal RNA genes belonging to *A.halanychi* showing closer genetic distances to *A.cornigera* than *A.bucera* (Table 2).

**Table 1.** Mitochondrial genome information for *Austropallene* species used in this study.

Species	Mitogeneome length (bp)	G-C content (%)	mtDNA genome
*Austropallenecornigera*	14,650 bp	21.60	OK623743
*Austropallenebucera*	15,177 bp	21.62	OK412987
*Austropallenehalanychi* sp. nov.	15,022 bp	21.10	OP781307

The mitochondrial genome information includes GenBank accession numbers, mitochondrial genome lengths, and G-C content for *Austropallenehalanychi*, *A.cornigera* and *A.bucera*.

**Table 2.** Genetic distances (calculated as uncorrected *p*-distances (%)) for mitochondrial protein-coding and ribosomal RNA genes between *Austropallenehalanychi* sp. nov., *A.cornigera*, and *A.bucera*.

Genetic distance (calclulated as uncorrected *p*-distance (%))			
Gene	*A.halanychi* and *A.cornigera*	*A.halanychi* and *A.bucera*	*A.cornigera* and *A.bucera*
ATP6	2.56	2.94	3.10
ATP8	2.86	5.52	2.76
COI	2.19	4.48	2.96
CO2	2.94	3.8	3.36
CO3	2.56	5.13	4.49
ND1	2.19	3.01	3.85
ND2	2.29	4.17	3.85
ND3	2.59	2.03	3.48
ND4	1.23	2.47	2.40
ND4L	1.81	3.26	2.90
ND5	1.85	3.05	2.93
ND6	1.69	2.53	2.95
CYTB	1.73	4.83	4.28
12S	1.32	2.12	1.171
16S	0.591	1.184	0.672

**Figure 6.**
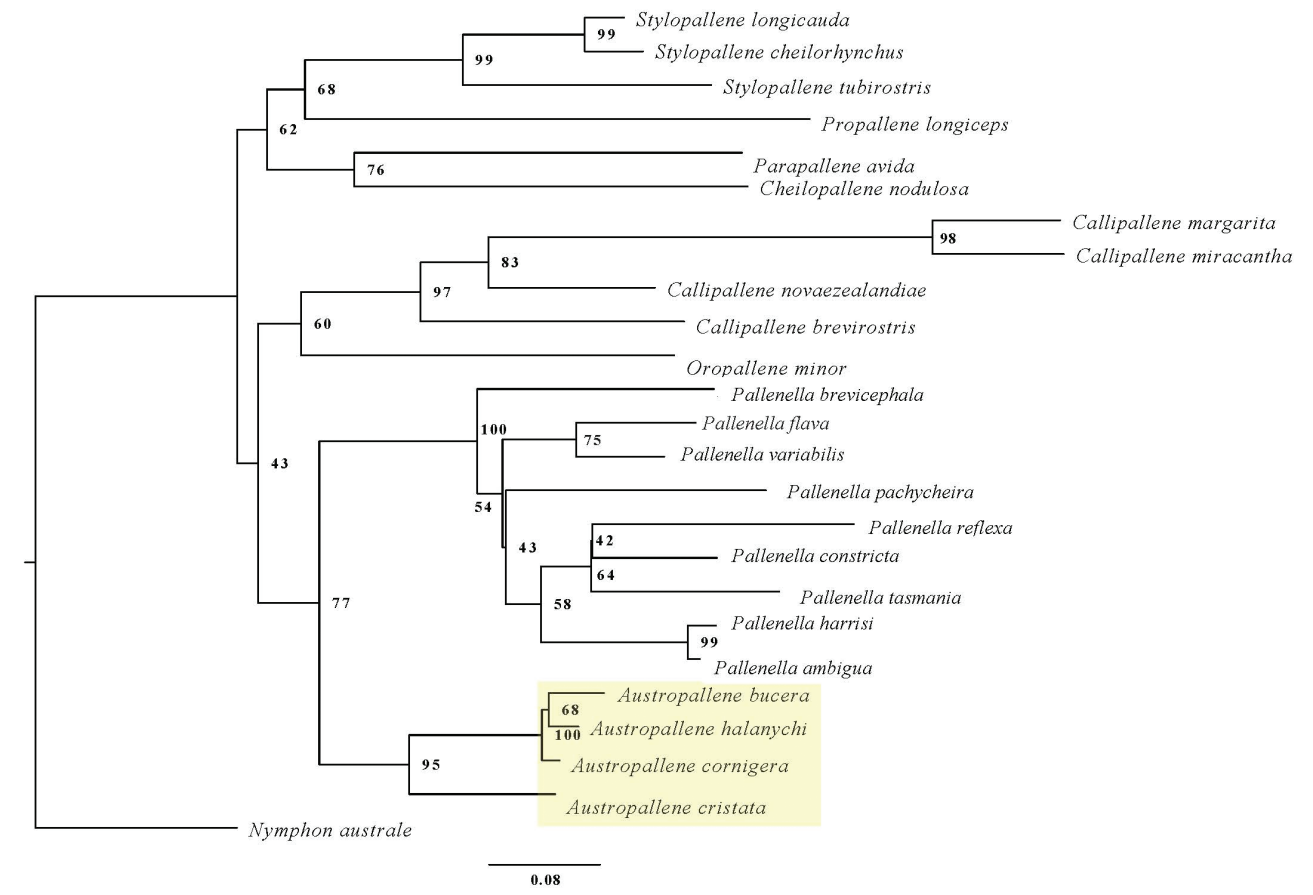
Phylogenetic tree constructed with partial COI data from members of the family Callipallenidae. The phylogenetic tree was constructed with IQ-TREE2 using a maximum likelihood approach and the best-fit model (GTR+F+I+G4). The clade containing *Austropallenehalanychi* sp. nov. is highlighted, and bootstrap support (based on 1000 rapid bootstrap replicates) is included.

The phylogenetic tree constructed using partial COI data available for members within Callipallenidae (Fig. 6) recovered a clade containing all *Austropallene* species placed as sister to a clade containing representatives of the genus *Pallenella* Schimkewitsch, 1909 (BS = 77). *Austropallenehalanychi* was recovered as sister to *A.bucera* (BS = 68), with this grouping placed as sister to *A.cornigera* (BS = 100). The clade containing *A.halanychi*, *A.bucera*, and *A.cornigera* was recovered as sister to *A.cristata* with strong support (BS = 95). The *Austropallene* + *Pallenella* clade was grouped as sister (BS = 43) to a clade containing representatives belonging to the genus *Callipallene* Flynn, 1929 in a monophyletic clade (BS = 97) recovered as sister to *Oropallene* Schimkewitsch, 1930 (BS = 60). Representatives belonging to the genus *Stylopallene* Clark, 1963 were recovered in a monophyletic clade (BS = 99) and placed as sister to *Propallene* Schimkewitsch, 1909 (Fig. 6). Representatives from the genera *Parapallene* Carpenter, 1892 and *Cheilopallene* Stock, 1955 were recovered as a monophyletic clade (BS = 76) placed as sister to the recovered *Stylopallene* + *Propallene* clade (BS = 62) (Fig. 6).

### ﻿*Austropallenehalanychi* sp. nov. in respect to closely related Antarctic *Austropallene* species

*Austropallenehalanychi* sp. nov. presents an arrangement and combination of morphological characters that have not been observed in any previously described *Austropallene* species (Table 3). We compared *A.halanychi* to three closely related and morphologically similar species of *Austropallene*: *A.cornigera* (Möbius, 1902), *A.bucera* Pushkin, 1993, and *A.tenuicornis* Pushkin, 1993. To begin, *A.halanychi* is larger and longer than the other closely related species, measuring 10.50 mm in body length, longer than *A.cornigera* (4.8 mm), *A.bucera*, (4.8 mm in male), and *A.tenuicornis* (4.5 mm) (Pushkin 1993). The length of the third walking legs in *A.halanychi* is 30.9 mm in length, longer than *A.cornigera* (22.1 mm) and *A.tenuicornis* (24.1 mm) but shorter than *A.bucera* (40.2 mm) (Pushkin 1993). One of the most distinctive characters that distinguish *A.halanychi* from these species are the powerful, relatively large, oblong chelifores. The chelifores in *A.halanychi* are longer and larger than in these species, with the scape measuring 2.40 mm and the claw measuring 3.37 mm in length, and differing from the more oval-shaped chelifores in *A.cornigera* (scape 2.2 mm, claw 2.6 mm), *A.bucera* (scape 2.0 mm, claw 2.4 mm), and *A.tenuicornis* (scape 2.30 mm, claw 2.3 mm) (Pushkin 1993). Furthermore, the fixed chelifore fingers of *A.halanychi* do not contain any denticles or notches on the inner surface, differing from *A.cornigera*, *A.bucera*, and *A.tenuicornis* all of which have denticles on the fixed finger (Table 3; Pushkin 1993). Additionally, the movable finger of the chelifore claw is distinctly shorter than the fixed finger of chelifore claw in *A.halanychi*, whereas the movable finger in *A.cornigera*, *A.bucera*, and *A.tenuicornis* is almost as long as the fixed finger (Table 3). The chelifore claw in *A.halanychi* closes completely when the fingers converge with no visible space remaining between the fixed and movable fingers; this differs from the new species’ congeners. The chelifore claw has two small, conical outgrowths on each side at the base where the chelifore fingers meet, a characteristic that is shared with *A.cornigera* but not with *A.bucera* or *A.tenuicornis* (Table 3). Moreover, the first coxa of the walking legs of *A.halanychi* contains small spines and setae, similar to *A.cornigera* and *A.tenuicornis*, but differing from *A.bucera* where the first coxa is smooth (Pushkin 1993). The listed similarities and differences distinguish *A.halanychi* from other similar species in the genus.

**Table 3.** Diagnostic characteristics for the genus *Austropallene*. The diagnostic characteristics belonging to the genus *Austropallene*, as well as known distribution locations for every Antarctic *Austropallene* species as described by Munilla and Soler Membrives (2008) with the addition of *Austropallenehalanychi* sp. nov. *Austropallenelukini* was not included here since it is not Antarctic in distribution.

Species	Trunk segments on dorsal side smooth and without spines	Cephalic spurs present	Presence of terminal spines on 10^th^ segment of ovigerous leg	Well pronounced expansion (heel) on the propodus of all walking legs	Tubercles with a bristle on the apex on the first coxa of walking legs	Cement glands present on femur in male	Denticle present in the inner surface of the fixed finger on the chelifore claw	Fixed finger of the chelifore claw (oval or pointed)	Conical outgrowths where fixed finger meets movable finger on dorsal and ventral sides	Movable finger of chelifore claw shorter than fixed finger	Tips of fingers converge when closed	No proximal gap observed when chelifore fingers close	Tubercles and setae present on chelifore scapes and claws	Known distribution locations
*Austropallenebrachyura*	X	X	X			X		Oval			X			Circumpolar
*Austropallenebucera*	X	X	X	X		X	X	Pointed		X				Scotia Sea–Subantarctic waters
*Austropallenecalmani*	X	X	X			X		Pointed		X			X	Circumpolar
*Austropallenecornigera*	X	X	X	X	X	X	X	Pointed			X			Circumpolar
*Austropallenecristata*		X	X	X		X		Pointed			X			Circumpolar
*Austropallenegracilipes*	X	X	X			X		Pointed			X			Scotia Sea–Subantarctic waters
*Austropallenehalanychi* sp.nov.	X	X	X	X	X	X		Pointed	X	X	X	X	X	Ross Sea
*Austropallenespinicornis*	X	X	X	X		X	X	Pointed			X			Scotia Sea–Subantarctic waters
*Austropallenetcherniai*	X	X	X			X		Pointed			X			Circumpolar
*Austropallenetibicina*	X	X	X			X		Pointed		X				Ross Sea and Scotia Sea
*Austropallenetenuicornis*	X	X	X	X			X	Pointed			X		X	Subantarctic Waters

## ﻿Conclusion

Morphologically, the comparably larger, more robust, and oblong chelifores and the absence of space between chelifore fingers, unequal length of chelifore fingers, absence of a denticle on either the movable or fixed chelifore claws, and ability to fully close chelifore fingers together all serve to distinguish this large individual from previously described species of *Austropallene*. Furthermore, the complete mitochondrial genome of *A.halanychi* sp. nov. follows the same distinctive gene order and directionality found in other members of the genus. The phylogenetic tree resulting from protein-coding gene data placed *A.halanychi* as sister to *A.cornigera* with the grouping in a clade with *A.bucera*. Furthermore, the phylogenetic tree resulting from partial COI data for members belonging to the Callipallenidae placed *A.halanychi* in a monophyletic clade with *A.cornigera*, *A.bucera*, and *A.cristata*, with this clade recovered as sister to a clade comprised of representatives from the genus *Pallenella*; this supports the placement of the new species in *Austropallene*. *Austropallenehalanychi* shares many morphological characteristics with *A.cornigera* (Table 3), indicating that these two species are closely related to one another. Overall, the combination of morphological characteristics and molecular evidence leaves no doubt that this is a new Antarctic species of *Austropallene*.

## Supplementary Material

XML Treatment for Austropallene halanychi

## ﻿Additional information

### Author ORCIDs

Jessica R. Zehnpfennig https://orcid.org/0000-0001-9865-2485

Andrew R. Mahon https://orcid.org/0000-0002-5074-8725

### Data availability

All of the data that support the findings of this study are available in the main text or Supplementary Information. The mitochondrial genome of *Austropallenehalanychi* sp. nov. is deposited in GenBank (https://www.ncbi.nlm.nih.gov/genbank/; accession number OP781307).

## Supplementary materials

Supplementary material 1 All species used in phylogenetic analyses for this study, which phylogenetic analysis the data was used for, and the correlated GenBank accession numbers Data type docx https://binary.pensoft.net/file/943970This dataset is made available under the Open Database License (http://opendatacommons.org/licenses/odbl/1.0/). The Open Database License (ODbL) is a license agreement intended to allow users to freely share, modify, and use this Dataset while maintaining this same freedom for others, provided that the original source and author(s) are credited.Jessica R. Zehnpfennig, Andrew R. Mahon

Supplementary material 2 Mitochondrial gene order of *Austropallenehalanychi* sp. nov. Data type pdf Explanation note The mitochondrial gene order, excluding introns, and the control region for *A.halanychi* and other members within Callipallenidae and Nymphonidae, as well as other families within Pycnogonida. The underlined segments represent genes found on the reverse strand of the mtDNA. https://binary.pensoft.net/file/943971This dataset is made available under the Open Database License (http://opendatacommons.org/licenses/odbl/1.0/). The Open Database License (ODbL) is a license agreement intended to allow users to freely share, modify, and use this Dataset while maintaining this same freedom for others, provided that the original source and author(s) are credited.Jessica R. Zehnpfennig, Andrew R. Mahon
