# Supplementary material for: ﻿Austropallenehalanychi sp. nov., a new species of sea spider (Pycnogonida, Callipallenidae) from the Ross Sea, Antarctica
Source: Zookeys. 2023 Nov 28;1185:163–80. doi: 10.3897/zookeys.1185.108286 (PMC10698867; doi:10.3897/zookeys.1185.108286)
Supplement: Supplementary material 1 — All species used in phylogenetic analyses for this study, which phylogenetic analysis the data was used for, and the correlated GenBank accession numbers [file zookeys-1185-163_article-108286__-s001.docx]

**Supplemental Table 1.** All species used in phylogenetic analyses for this study, which phylogenetic analysis the data was used for, and the correlated GenBank accession numbers.

| Species | Analysis | GenBank accession(s) |
| --- | --- | --- |
| *Ammothea calmani* | Mitochondrial | OK583907 |
| *Ammothea carolinensis* | Mitochondrial | GU065293.1/NC_014671.1 |
| *Ammothea hilgendorfi* | Mitochondrial | GU370075 |
| *Achelia bituberculata* | Mitochondrial | AY457170.1 |
| *Tanystylum orbiculare* | Mitochondrial | GU370074.1/NC_014505.1 |
| *Tanystylum sp.* | Mitochondrial | OK649925 |
| *Ammothea clausi* | Mitochondrial | OK573458 |
| *Austrodecus sp.* | Mitochondrial | OK623745 |
| *Austropallene cornigera* | Mitochondrial/COI | OK623743 |
| *Austropallene bucera* | Mitochondrial/COI | OK412987 |
| *Austropallene halanychi* ***sp.nov.*** | Mitochondrial/COI | OP781307 |
| *Colossendeis megalonyx* | Mitochondrial | HQ450773.1 |
| *Decolopoda australis* | Mitochondrial | OK623744 |
| *Colossendeis brevirostris* | Mitochondrial | OK623747 |
| *Colossendeis robusta* | Mitochondrial | OK649923 |
| *Endeis sp. #1* | Mitochondrial | OK649926 |
| *Endeis sp. #2* | Mitochondrial | OK649924 |
| *Pallenopsis pilosa* | Mitochondrial | OK649918 |
| *Pallenopsis patagonica* | Mitochondrial | OK649919 |
| *Anoplodactylus australis* | Mitochondrial | OK649922 |
| *Pycnogonum diceros* | Mitochondrial | OK649921 |
| *Pentapycnon charcoti* | Mitochondrial | OK649920 |
| *Rhynchothorax sp. #1* | Mitochondrial | OK649914 |
| *Rhynchothorax sp. #2* | Mitochondrial | OK649915 |
| *Nymphon brevicaudatum* | Mitochondrial | OK649917 |
| *Nymphon australe* | Mitochondrial/COI | OK649916 |
| *Nymphon gracile* | Mitochondrial | DQ666063.1 |
| *Nymphonella tapetis* | Mitochondrial | MT864846.1, MT865030.1, MT864980.1, MT865096.1, MT865187.1, MT865244.1, MT865310.1, MT865452.1 |
| *Ascorhynchus abyssi* | Mitochondrial | MT864844.1, MT865015.1, MT864968.1, MT864910.1, MT865098.1, MT865246.1, MT865337.1, MT865458.1 |
| *Eurycyde hispida* | Mitochondrial | MT865044.1, MT864971.1, MT864901.1, MT865101.1, MT865190.1, MT865331.1, MT865317.1, MT865410.1 |
| *Austropallene cristata* | COI | DQ390045.1 |
| *Callipallene brevirostris* | COI | MK308207.1 |
| *Pallenella ambigua* | COI | MT865061.1 |
| *Pallenella brevicephala* | COI | HQ970319.2 |
| *Pallenella constricta* | COI | HQ970314.2 |
| *Pallenella flava* | COI | HQ970313.2 |
| *Pallenella tasmania* | COI | HM432456.1 |
| *Pallenella variabilis* | COI | HM381706.1 |
| *Pallenella harrisi* | COI | HM381709.1 |
| *Pallenella reflexa* | COI | MT865068.1 |
| *Pallenella pachycheira* | COI | MT865073.1 |
| *Stylopallene cheilorhynchus* | COI | HM432451.1 |
| *Stylopallene tubirostris* | COI | HM381708.1 |
| *Cheilopallene nodulosa* | COI | KX535430.1 |
| *Callipallene margarita* | COI | KF603927.1 |
| *Callipallene miracantha* | COI | MT865062.1 |
| *Callipallene novaezealandiae* | COI | DQ390082.1 |
| *Stylopallene longicauda* | COI | DQ390084.1 |
| *Parapallene avida* | COI | DQ390083.1 |
| *Oropallene minor* | COI | DQ390059.1 |
| *Propallene longiceps* | COI | DQ390054.1 |
