# Supplementary material for: ﻿Austropallenehalanychi sp. nov., a new species of sea spider (Pycnogonida, Callipallenidae) from the Ross Sea, Antarctica
Source: Zookeys. 2023 Nov 28;1185:163–80. doi: 10.3897/zookeys.1185.108286 (PMC10698867; doi:10.3897/zookeys.1185.108286)
Supplement: Supplementary material 2 — Mitochondrial gene order of Austropallenehalanychi sp. nov. [file zookeys-1185-163_article-108286__-s002.pdf]

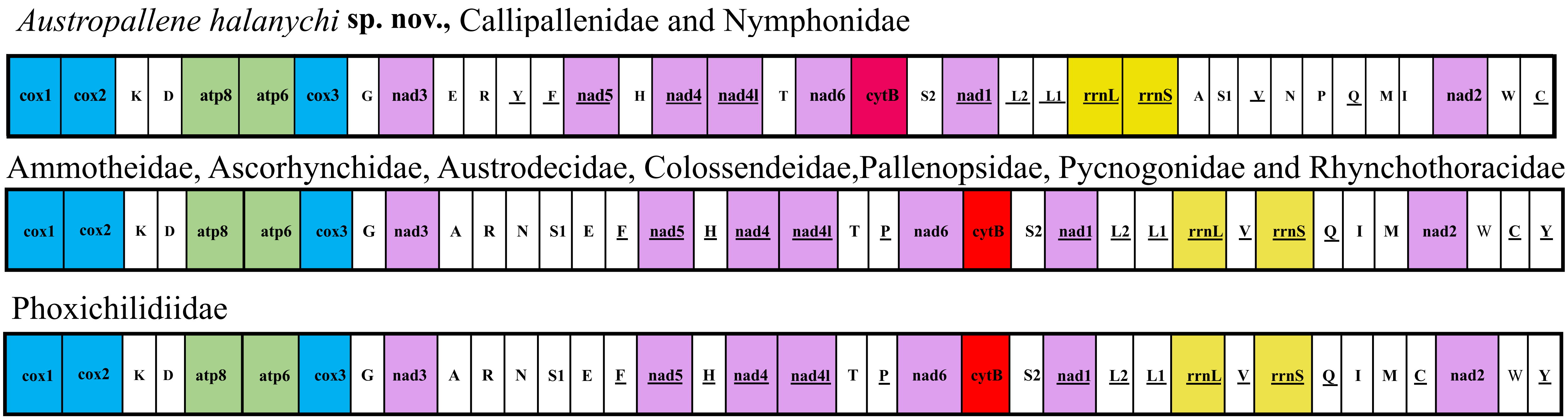
Austropallene halanychi **sp. nov.**, Callipallenidae and Nymphonidae
cox1 cox2 K D atp8 atp6 cox3 G nad3 E R Y F nad5 H nad4 nad4l T nad6 cytB S2 nad1 L2 L1 rrnL rrnS A S1 V N P Q M I nad2 W C
Ammotheidae, Ascorhynchidae, Austrodecidae, Colossendeidae,Pallenopsidae, Pycnogonidae and Rhynchothoracidae
cox1 cox2 K D atp8 atp6 cox3 G nad3 A R N S1 E F nad5 H nad4 nad4l T P nad6 cytB S2 nad1 L2 L1 rrnL V rrnS Q I M nad2 W C Y
Phoxichilidiidae
cox1 cox2 K D atp8 atp6 cox3 G nad3 A R N S1 E F nad5 H nad4 nad4l T P nad6 cytB S2 nad1 L2 L1 rrnL V rrnS Q I M C nad2 W Y
